# Raman Microspectroscopy Detection and Characterisation of Microplastics in Human Breastmilk

**DOI:** 10.3390/polym14132700

**Published:** 2022-06-30

**Authors:** Antonio Ragusa, Valentina Notarstefano, Alessandro Svelato, Alessia Belloni, Giorgia Gioacchini, Christine Blondeel, Emma Zucchelli, Caterina De Luca, Sara D’Avino, Alessandra Gulotta, Oliana Carnevali, Elisabetta Giorgini

**Affiliations:** 1 Department of Obstetrics and Gynecology, Università Campus Bio Medico di Roma, Via Alvaro del Portillo, 200, 00128 Roma, Italy; antonio.ragusa@gmail.com; 2 Department of Life and Environmental Sciences, Università Politecnica delle Marche, Via Brecce Bianche, 60131 Ancona, Italy; a.belloni@pm.univpm.it (A.B.); giorgia.gioacchini@univpm.it (G.G.); o.carnevali@univpm.it (O.C.); e.giorgini@univpm.it (E.G.); 3 Department of Obstetrics and Gynecology, San Giovanni Calibita Fatebenefratelli Hospital, Isola Tiberina, Via di Ponte Quattro Capi, 39, 00186 Roma, Italy; alessandrosvelato@virgilio.it (A.S.); christineblondeel@yahoo.fr (C.B.); emmazucchelli16@gmail.com (E.Z.); deluca.caterina33@gmail.com (C.D.L.); saradavino@libero.it (S.D.); 4 Department of Obstetrics and Gynecology, Università degli Studi di Sassari, Viale S. Pietro, 07100 Sassari, Italy; alessandragulotta87@gmail.com

**Keywords:** microplastics, human breastmilk, Raman microspectroscopy, infants’ nutrition

## Abstract

The widespread use of plastics determines the inevitable human exposure to its by-products, including microplastics (MPs), which enter the human organism mainly by ingestion, inhalation, and dermal contact. Once internalised, MPs may pass across cell membranes and translocate to different body sites, triggering specific cellular mechanisms. Hence, the potential health impairment caused by the internalisation and accumulation of MPs is of prime concern, as confirmed by numerous studies reporting evident toxic effects in various animal models, marine organisms, and human cell lines. In this pilot single-centre observational prospective study, human breastmilk samples collected from N. 34 women were analysed by Raman Microspectroscopy, and, for the first time, MP contamination was found in 26 out of 34 samples. The detected microparticles were classified according to their shape, colour, dimensions, and chemical composition. The most abundant MPs were composed of polyethylene, polyvinyl chloride, and polypropylene, with sizes ranging from 2 to 12 µm. MP data were statistically analysed in relation to specific patients’ data (age, use of personal care products containing plastic compounds, and consumption of fish/shellfish, beverages, and food in plastic packaging), but no significant relationship was found, suggesting that the ubiquitous MP presence makes human exposure inevitable.

## 1. Introduction

The global production of plastics has reached the impressive amount of more than 350 million tons per year. This is the result of the massive demand for this material, which has been considered, until now, the golden choice in terms of durability, usability, and versatility for a huge variety of applications and consumer products [1,2,3]. This widespread use of plastics also led to their accumulation in landfills and in the natural environment. In fact, as a consequence of the extensive production and employment of single-use products, which represent >40% of manufactured plastics, 250,000 tons of plastic litter is estimated to be floating in the oceans [4]. Although several countries are introducing new regulations on plastic waste management and recycling strategies, it has to be noted that in 2018 in Europe, only 32.5% of post-consumer waste plastic was recycled, while 24.9% was accumulated in landfills [5].

Environmental plastic contamination derives from several factors, including mismanaged plastic waste, fishing nets in the sea, and different household and commercial activities, such as washing synthetic textiles, road markings, tires, marine coatings, personal care products, and plastic pellets [6,7]. In particular, after being released into the environment, plastic products undergo a degradation process caused by the action of atmospheric agents, such as waves, abrasion, UV radiation, and photo-oxidation, in combination with biological processes, which leads to the formation of microplastics (MPs) [8]. MPs range from 5 millimetres to 100 nanometres and are classified as primary or secondary based on their source of release into the environment: primary MPs are purposely manufactured at sizes <5 mm to be employed for commercial purposes (such as glitter in cosmetic products and microbeads in cleansers, scrubs, and dish scrubbing pads), while secondary MPs are generated by the previously described environmental degradation processes of larger plastic items [9,10].

The ubiquitous occurrence of MPs in the environment determines inevitable human exposure, mainly by three routes: ingestion, inhalation, and dermal contact. Among all of them, ingestion is considered the major route, with an estimated intake of 39 ÷ 52 thousand MPs per person per year [11,12,13]. Once internalised, MPs may pass across cell membranes [14], followed by accumulation or elimination by the onset of specific cellular mechanisms. All of these processes are mainly related to MPs’ size, which cannot exceed 10–15 μm.

The potential health impairment caused by the internalisation and accumulation of MPs is of prime concern. Although information is still lacking on this topic, several studies reported evident toxic effects in various animal models, marine organisms, and human cell lines [15,16,17], showing that MPs, once internalised, are not inert as previously supposed and likely trigger local or systemic responses.

Given the strong concern related to the effects of MPs on animal and human health, the use of reliable and objective techniques for MP detection and characterisation is crucial. Among all of the exploited techniques, Raman Microspectroscopy (RMS) can be considered the gold standard, since it lets researchers characterise not only the morphological features of microparticles but also their chemical composition in terms of both polymer matrices and pigments. Moreover, RMS presents the advantage of enabling the analysis of MPs as small as ~2 µm directly on filtration membranes, thanks to the high potential of light scattering [18,19,20]. Recently, our research group, for the first time, detected the presence of MPs in human placenta samples; this study, carried out by Raman Microspectroscopy, received extensive attention, since the delicate role played by this organ may be perturbed by the presence of MPs [21].

Based on these impressive results, we decided to investigate the contamination of microplastics in breastmilk to assess another MP exposure route in the extremely vulnerable population of infants. For this purpose, in the present study, milk samples collected from 34 consenting patients were analysed by Raman Microspectroscopy, and, for the first time, in most of the analysed samples, the presence of MPs was detected. The relevance of this research lies in breastmilk being the gold standard for infants’ nutrition. Moreover, it reflects both the mother’s and infant’s postnatal exposure, and hence, it represents an optimal matrix for contaminant biomonitoring [22]. In fact, milk consists of protein and fat globules in a carbohydrate-based suspension and represents a favourable environment for the lipophilic nature of MPs and other chemicals. In this regard, it is noteworthy that several studies reported the contamination of breastmilk by phthalates, heavy metals, and perfluorinated compounds [23,24,25].

## 2. Materials and Methods

### 2.1. Cohort Selection

This was a pilot observational descriptive study in a prospective and single-centre cohort. It was approved by the Ethical Committee Lazio 1 (Protocol N. 708/CE Lazio 1; 24 May 2021), and it was carried out in full accordance with ethical principles, including The Code of Ethics of the World Medical Association (Declaration of Helsinki) for experiments involving humans. A dedicated cohort of N. 34 patients, all characterised by pregnancies without complications, was enrolled at Ospedale Fatebenefratelli Isola Tiberina (Rome, Italy). Exclusion criteria were: (a) medically prescribed special diets within 4 weeks prior to delivery; (b) diarrhoea or severe constipation within 2 weeks prior to delivery; (c) use of antibiotics within 2 weeks prior to delivery; (d) use of drugs affecting intestinal resorption, e.g., activated charcoal or cholestyramine, within 2 weeks prior to delivery; (e) diagnosis of a gastrointestinal pathology, e.g., ulcerative colitis or Crohn’s disease, except for appendectomy; (f) cancer, HIV, or any other serious illness demanding medical treatment; (g) invasive or abrasive dental treatments within 2 weeks prior to delivery; (h) alcohol abuse (defined as Alcohol Use Disorder Identification test >10); and (i) current or recent (within the previous 4 weeks) participation in a clinical trial. Patients who decided to participate were asked to sign an informed consent form. Patients were also asked to fill in a questionnaire to record their food consumption, with a special focus on fish, shellfish, and foods employing packaging, and the use of personal care products from 7 days before the expected date of delivery to 7 days after.

### 2.2. Sample Collection

Breastmilk samples were collected 1 week after delivery at the Department of Obstetrics and Gynaecology of San Giovanni Calibita Fatebenefratelli Hospital (Rome, Italy). Patients were guided on a manual expression procedure, recommended by the World Health Organization and described in a document released by the Italian Ministry of Health [26], which uses manual expression to obtain the maximum milk output and to avoid pain or damage to the breast tissue. No breast pumps were allowed to avoid contamination from its plastic components. Briefly, the manual expression procedure consists of cupping the breast with one hand, with the other forming a C-shape with the thumb and the forefinger, 3–4 cm from the base of the nipple; then, pressure is applied by pushing towards the ribcage, squeezing with the thumb and forefinger, and finally releasing the pressure. The sequence of pressure, squeeze, and release was repeated until obtaining an adequate amount of milk. Milk samples were placed into glass flasks, weighed, and then stored at −20 °C until processing. Each sample contained an average amount of 4.16 ± 1.73 g of breastmilk.

### 2.3. Sample Digestion and Filtration

In order to remove organic components from milk samples, a digestion protocol was set up and performed at the Laboratory of Vibrational Spectroscopy, Department of Life and Environmental Sciences, Università Politecnica delle Marche (Ancona, Italy). A 10% KOH solution prepared using 1.6 µm filtered deionised water and KOH tablets (Sigma-Aldrich) was added to each flask in a ratio of sample to KOH of 1:10 (*w*/*v*). Flasks were sealed and incubated at 40 °C for 48 h [27]. Digestates were then filtered through a 1.6 µm pore-size filter membrane (Whatman GF/A) by a vacuum pump connected to a filter funnel. Filter membranes were dried at room temperature and stored in glass Petri dishes until Raman Microspectroscopy (RMS) analysis.

### 2.4. Detection and Identification of MPs by Raman Microspectroscopy

RMS analysis was performed by using an XploRA Nano Raman Microspectrometer (Horiba Scientific) at the ARI Laboratory of Università Politecnica delle Marche (Ancona, Italy). All filter membranes, including those deriving from the procedural blanks, were inspected by visible light using a ×10 objective (Olympus MPLAN10×/0.25). The detected MPs were morphologically characterised by a ×100 objective (Olympus MPLAN100×/0.90) and then directly analysed on the filter by RMS (spectral range 200–1800 cm^−1^, 532 nm or 785 nm laser diode, 600 lines per mm grating). Spectra were dispersed onto a 16-bit dynamic range Peltier-cooled CCD detector; the spectrometer was calibrated to the 520.7 cm^−1^ line of silicon prior to spectral acquisition. To reduce noise and enhance spectrum quality, raw Raman spectra were subjected to polynomial baseline correction and vector normalisation (Labspec 6 software, Horiba Scientific). The polymer matrix of the detected particles was identified by comparing the collected Raman spectra with spectral libraries of polymers and pigments obtained by measuring standard polymers/compounds (KnowItAll software, John Wiley & Sons, Inc., Hoboken, NJ, USA) [28,29]. Similarities of more than 80 of the Hit Quality Index (HQI) were considered satisfactory.

### 2.5. Quality Assurance and Control (QA/QC)

Efforts were adopted to avoid microplastic contamination during sample collection, storage, processing, and analysis. To this aim, a plastic-free protocol was adopted during all phases of the experiment, and a dedicated room was used for the digestion of milk samples, filtration, and RMS analysis steps. Routinely employed plastic tools were replaced with sterilised glass ones. Cotton laboratory coats and single-use latex gloves were worn during all phases of the experiment. All liquids, including ethanol for cleaning and deionised water for cleaning and preparation of all solutions, were filtered through 1.6 µm pore-size filter membranes (Whatman GF/A). Work surfaces were thoroughly washed with 70% ethanol prior to starting all procedures and during the experimental time. Glassware and instruments, including scissors and tweezers, were washed using dishwashing liquid, triple rinsed with 70% ethanol, and finally rinsed with 1.6 µm filtered deionised water.

Moreover, environmental and procedural blanks were prepared and thoroughly analysed to detect microplastic contamination deriving from the laboratory environment and from other external sources. As regards environmental blanks, a filter membrane soaked with 1.6 µm filtered deionised water was placed into an uncovered Petri dish and positioned each day in the above-mentioned dedicated room. A procedural blank was also prepared together with every batch of samples following the exact same procedure as samples, but without adding milk. The filters deriving from environmental and procedural blanks were first inspected by stereomicroscope.

### 2.6. Statistical Analysis

Data analysis was performed by using the statistical software package Prism6 (Graphpad Software, Inc., San Diego, CA, USA). Normality was checked by the D’Agostino and Pearson omnibus normality test. Chi-square test, Student’s *t*-test, and one-way analysis of variance (ANOVA) were performed to compare data accordingly. The significance threshold was set at *p* < 0.05.

## 3. Results

In the present study, N. 34 breastmilk samples were investigated by RMS for the presence of microplastics. MP contamination was found in 26 out of 34 women. As regards QA/QC protocols, the analysis of environmental (N. 14) and procedural (N. 9) blanks was performed. In the environmental blanks, only fibres, for a total of N. 16, ranging from 571 µm to 3000 µm, were found. Conversely, no MP contamination was found in the filters from the procedural blanks. Given the dimensions of the fibres, which are not compatible with the translocation into breastmilk, and the absence of fibres in the analysed milk samples, there was no need to blank-correct the results.

All details about the identified microparticles (such as morphology, dimensions, colour, polymer matrix, and pigment) are listed in Table 1.

**Figure 1:**
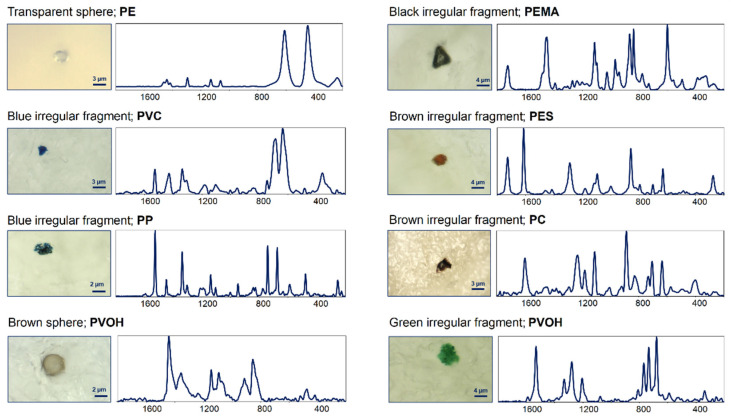
Microphotographs and Raman spectra (wavenumbers, cm^−1^) of some selected MPs found in the analysed breastmilk samples. PE: polyethylene; PVC: polyvinyl chloride; PP: polypropylene; PVOH: polyvinyl alcohol; PEVA: poly(ethylene-co-vinyl acetate); PEMA: poly(ethyl methacrylate); PES: polyester, and PC: polycarbonate.

For clarity, in Figure 1, the microphotographs and corresponding Raman spectra of some selected MPs found in the analysed samples are reported.

**Figure 2:**
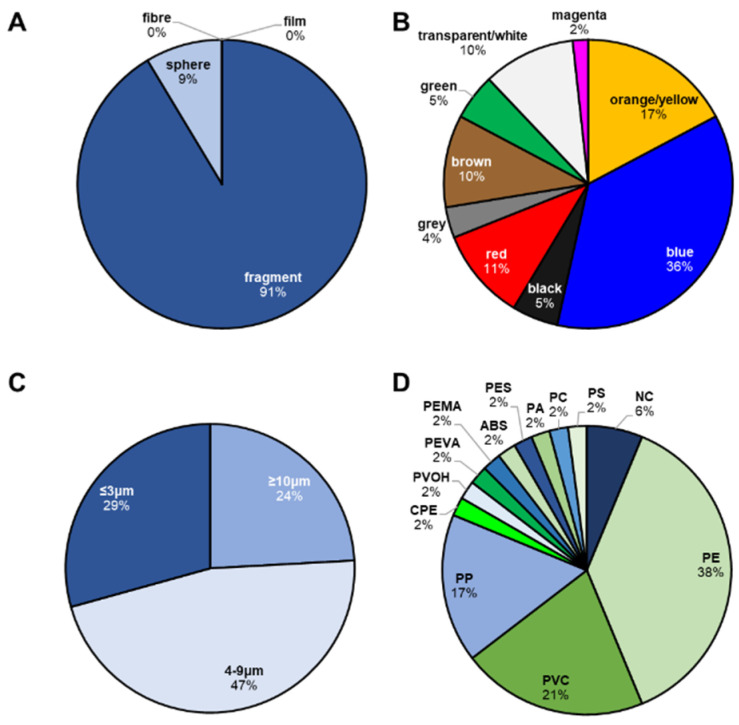
Percentage abundances of identified shapes (**A**), colours (**B**), dimensions (**C**), and polymer matrices (**D**). PE: polyethylene; PVC: polyvinyl chloride; PP: polypropylene; CPE: chlorinated polyethylene; PVOH: polyvinyl alcohol; PEVA: poly(ethylene-co-vinyl acetate); PEMA: poly(ethyl methacrylate); ABS: acrylonitrile butadiene styrene; PES: polyester; PA: polyamide; PC: polycarbonate; PS: polystyrene, and NC: nitrocellulose.

The detected microparticles were classified according to their shape, colour, dimensions, and chemical composition (Figure 2). As regards the shape, only irregular fragments and spheres were found, while no films or fibres were identified (Figure 2A). Moreover, most of the identified MPs were pigmented (ca. 90%), with blue and orange/yellow being the most abundant colours (ca. 36% and ca. 17%, respectively; Figure 2B). As regards MPs’ dimensions, almost half of them (ca. 47%) were in the range of 4–9 µm; ca. 29% were ≤3 µm, while ca. 24% were ≥10 µm (Figure 2C). For 48 out of the total 58 identified MPs, the polymer matrix was also identified, while for the remaining ones, the contribution of the pigments used for plastic staining was the only signal in the collected Raman spectra [21,30,31]. Within the identified polymer matrices, the most abundant ones were polyethylene (PE, 38%), polyvinyl chloride (PVC, 21%), and polypropylene (PP, 17%) (Figure 2D).

**Figure 3:**
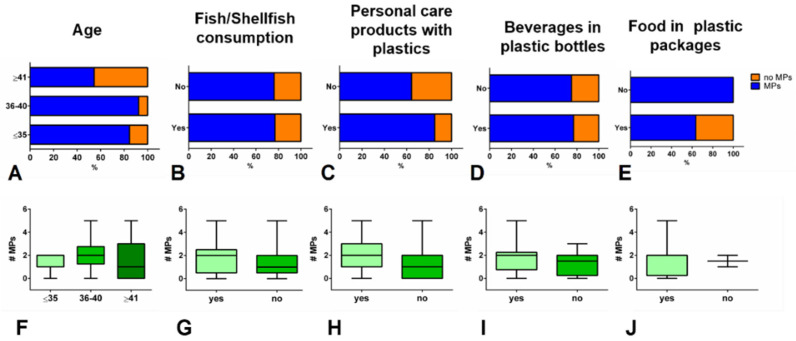
Percentage abundances of samples with (MPs) and without (no MPs) microplastics, divided according to the following selected parameters: (**A**) age of patient; (**B**) consumption of fish/shellfish in the 7 days prior to the expected date of delivery and 7 days after; (**C**) use of personal care products with plastic compounds in the 7 days prior to the expected date of delivery and 7 days after; (**D**) consumption of beverages in plastic bottles in the 7 days prior to the expected date of delivery and 7 days after, and (**E**) consumption of food in plastic packaging in the 7 days prior to the expected date of delivery and 7 days after. Number of identified microplastics divided according to the above-defined parameters (**F**–**J**) (box charts: centre line marks the median, edges indicate the 5th and 95th percentiles, and whiskers indicate the minimum and maximum values).

The statistical relationship between data related to patients and the presence/number of MPs was also investigated. In particular, the following patient-related parameters were analysed: patient’s age; use of personal care products containing plastic compounds (including lotions, soaps, and toothpaste); and consumption, in the 7 days prior to the expected date of delivery and 7 days after, of fish/shellfish, beverages in plastic bottles, and food in plastic packaging. In Figure 3A–E, the statistical analysis of the percentages of women with and without MPs in their milk, divided according to each of the above-defined parameters, is reported. As regards ‘patient’s age’, women were divided into three groups as follows: ≤35 years old, 36–40 years old, and ≥41 years old; no statistically significant difference was observed among groups (*p* = 0.648; Figure 3A). Moreover, women’s habits of food consumption and use of personal care products were investigated, and in this case as well, no statistically significant difference among groups was observed (‘fish/shellfish consumption’, *p* = 0.961, Figure 3B; ‘personal care product with plastics’, *p* = 0.1611, Figure 3C; ‘beverages in plastic bottles’, *p* = 0.9107, Figure 3D; and ‘food in plastic packaging’, *p* = 0.2963, Figure 3E). For a deeper analysis, each of the above-defined parameters was also considered in relation to the number of detected MPs (Figure 3F–J); as expected, no significant difference among groups was revealed (one-way ANOVA or Student’s *t*-test).

## 4. Discussion

Breastmilk represents the best standard nutrition for infants, thanks to its provision of nutrients and enhancement of the immune system [24]. Hence, assessing its quality in terms of possible contamination is mandatory. In fact, mothers are exposed daily to a great variety of chemicals present in the environment, for example, through food, beverages, and personal care products, and for this reason, breastmilk may be contaminated by these compounds, likely impacting children’s health [32]. To date, the presence of polychlorinated bisphenyls (PCBs), organochlorine pesticides, polybrominated diphenyl esters (PBDEs), phthalates and phthalate metabolites, per- and polyfluoroalkyl substances (PFASs), phenols, and metals have been detected in human milk [33]. In fact, since most of these contaminants are lipophilic and have a tendency to deposit in adipose tissue, they may be translocated to milk during lactation [34,35]. It is known that early life stages are the most sensitive to PCBs’ toxic effects, which mainly consist of a severe impact on endocrine and cognitive systems, leading to reduced IQ and altered behaviour [36]. Similarly, PBDEs are recognised as neurotoxic, especially in children, with effects on motor, cognitive, and behavioural development [37]. Phthalates have been reported in the literature to negatively impact male reproductive functionality [38]; moreover, childhood exposure to phthalates was shown to increase the risk of allergic diseases and altered physical/neurocognitive development [39,40].

It is noteworthy that most of these environmental pollutants are also able to interact with MPs by several sorption mechanisms, which depend on polymer size, shape, density, colour, and chemical composition [41]; for example, phthalate esters have shown major sorption on polystyrene, polyethylene, and polyvinyl chloride microparticles [42]. Hence, since MPs are ubiquitous environmental contaminants and represent potential vectors for toxic organic compounds with known health impairment effects, their detection in biological matrices is actually of great concern [15,16,17].

In this study, for the first time, MPs were found in breastmilk samples; it should be stated that the number of microparticles that we detected could be underestimated, since only an aliquot of ~4 g of milk was considered for each sample. MPs were characterised by RMS and classified in terms of shape, dimensions, colour, and chemical composition. In this regard, almost all MPs were blue and orange/yellow irregular fragments with dimensions ranging from 2 µm to 12 µm, consistent with translocation mechanisms. In accordance with other studies reported in the literature, the most abundant polymers were polyethylene, polyvinyl chloride, and polypropylene [43,44].

Several MP routes of exposure have been reported in the literature, including inhalation, dermal contact, and ingestion, with the latter being considered the most impactful, with an estimated total intake of around 39–52 thousand per person per year [12,13]. Once ingested/inhaled, MPs can be internalised in human tissues [11]. At the gastrointestinal level, they may pass through the epithelium by endocytosis mechanisms or by paracellular diffusion, after which they are translocated by dendritic cells through the lymphatic circulation and reach the circulatory system [11]. As regards the respiratory system, inhaled MPs likely penetrate the lower respiratory tract, characterised by a thin mucus layer, and spread into the bloodstream after cellular uptake or paracellular diffusion [45].

Currently, there is growing scientific evidence about MPs in humans. Schwabl et al. reported the detection of MPs in human stool [46], while, as a further measure, Ibrahim et al. described the presence of MPs in human colectomy samples, proving that MPs in part cross the intestinal barrier [47]. As evidence of inhalation exposure, Amato-Lourenço et al. detected, in human lung tissue, <5.5 μm polymeric MPs and fibres ranging from 8.12 to 16.8 μm [48]. We recently found MPs in the human placenta, which represents the interface between the foetus and the mother exposed to the external environment [21], results that were also confirmed by Braun et al. [49]. Very recently, the presence of plastic particles in human blood finally proved the transport of MPs in the bloodstream to every body site [50].

As regards the mammary gland, two hypothetical pathways have been suggested for the translocation of exogenous particles from the bloodstream to breast milk: the mammary epithelial cell-dependent and the immune cell-dependent pathways, with the latter being particularly relevant in the case of inhaled particles [23,24,25,51,52]. Hence, a possible association between the presence of MPs in breastmilk and specific information regarding mothers’ habits (such as the consumption of fish and shellfish, beverages in plastic bottles, and food in plastic packaging and the use of personal care products containing plastic compounds in the 7 days prior to the expected date of delivery and the 7 days after) was investigated, but no relationship was found between MP presence/number and each of the above-mentioned parameters.

The lack of association with the use of personal care products is likely explained by considering that dermal contact has a minor impact as an exposure route, since only particles <100 nanometres can cross the dermal barrier [11]. Conversely, the absence of a relation with mothers’ food habits is more difficult to explain, since the major route of MP exposure is represented by ingestion. In fact, numerous food-related sources of MPs have been reported, including fish, shellfish, and human essential daily consumables, such as table salt, sugar, bottled water, milk, honey, plastic teabags, and to a greater extent, plastic kitchen tools, plates, and packaging [53]. Hence, our findings suggest that, since MPs are ubiquitous in the environment, exposure to these microparticles is inevitable, and, for this reason, it is impossible to isolate a specific source among the complex set of faced exposures.

## 5. Conclusions

The evidence of MPs in human breastmilk, coupled with the previous discovery of these microparticles in the human placenta, represents a great concern, since it impacts the extremely vulnerable population of infants. In fact, the chemicals possibly contained in foods, beverages, and personal care products consumed by breastfeeding mothers may be transferred to the offspring, potentially exerting a toxic effect. Hence, it is mandatory to increase efforts in scientific research to deepen the knowledge of the potential health impairment caused by MP internalisation and accumulation, especially in infants, and to assess innovative, useful ways to reduce exposure to these contaminants during pregnancy and lactation.

## Figures and Tables

**Table 1:** Information about patients (age, quantity of milk sample, and abundance of MPs) and morphological and chemical features of the identified microparticles.

Patient	Age	Milk Quantity (g)	N. of MPs	MP/g	Shape	Size	Colour	Polymer Matrix	Pigment
#1	28	8.44	2	0.24	irregular fragment	~10 µm	orange	nitrocellulose	
					irregular fragment	~6 µm	orange	polyethylene	
#2	32	4.92	1	0.20	irregular fragment	~3 µm	blue	polyethylene	
#3	32	5.83	1	0.17	irregular fragment	~6 µm	black	polyvinyl chloride	
#4	38	5.09	2	0.39	irregular fragment	~2 µm	red	polyvinyl chloride	
					irregular fragment	~3 µm	blue	polypropylene	
#5	36	7.08	2	0.28	irregular fragment	~6 µm	red	chlorinated polyethylene	
					sphere	~5 µm	grey	polypropylene	
#6	40	1.95	4	2.05	irregular fragment	~5 µm	light blue	polyvinyl chloride	
					irregular fragment	~1 µm	blue		Pigment Blue 29 (C.I. Constitution 77007)
					irregular fragment	~10 µm	light blue		Pigment Green 7 (C.I. Constitution 74260)
					irregular fragment	~10 µm	light blue		Pigment Green 7 (C.I. Constitution 74260)
#7	38	3.49	5	1.43	sphere	~5 µm	brown	polyvinyl alcohol	
					irregular fragment	~3 µm	light blue		Pigment Green 7 (C.I. Constitution 74260)
					irregular fragment	~10 µm	brown/grey	nitrocellulose	
					sphere	~2 µm	blue		Pigment Blue 29 (C.I. Constitution 77007)
					irregular fragment	~10 µm	light blue	polypropylene	
#8	36	4.21	2	0.47	irregular fragment	~2 µm	red		Pigment Red 101/102 (C.I. Constitution 77491)
					irregular fragment	~10 µm	red		Pigment Red 101/102 (C.I. Constitution 77491)
#9	45	2.81	3	1.07	irregular fragment	~4 µm	red		Pigment Red 101/102 (C.I. Constitution 77491)
					irregular fragment	~5 µm	yellow/orange	polyethylene	
					irregular fragment	~3 µm	light blue	polyvinyl chloride	
#10	34	5.58	2	0.36	irregular fragment	~6 µm	orange	polyethylene	
					irregular fragment	~6 µm	blue	polypropylene	
#11	39	2.32	0	0					
#12	32	3.55	2	0.56	irregular fragment	~2 µm	black	polyethylene	
					irregular fragment	~10 µm	green	poly(ethylene-co-vinyl acetate)	Pigment Green 7 (C.I. Constitution 74260
#13	41	2.76	1	0.36	irregular fragment	~12 µm	blue	polyethylene	
#14	50	4.50	3	0.67	irregular fragment	~3 µm	brown		Pigment Yellow 43 / Brown 6 (C.I. Constitution 77492)
					irregular fragment	~3 µm	light blue	polyvinyl chloride	
					sphere	~4 µm	grey	polypropylene	
#15	33	5.99	2	0.33	irregular fragment	~5 µm	orange	polyethylene	
					irregular fragment	~6 µm	red	polyvinyl chloride	
#16	37	4.65	2	0.43	irregular fragment	~10 µm	blue	polyethylene	
					irregular fragment	~2 µm	transparent	polyethylene	
#17	32	1.64	2	1.22	sphere	~5 µm	transparent	polyethylene	
					irregular fragment	~5 µm	brown		Pigment Red 101/102 (C.I. Constitution 77491)
#18	41	3.10	1	0.32	irregular fragment	~8 µm	black	poly(ethyl methacrylate)	
#19	38	6.06	2	0.33	irregular fragment	~8 µm	orange	nitrocellulose	
					irregular fragment	~2 µm	blue/green	polypropylene	
#20	37	4.19	3	0.71	irregular fragment	~3 µm	magenta	polyvinyl chloride	
					irregular fragment	~12 µm	light blue	polyvinyl chloride	
					irregular fragment	~12 µm	green	polyvinyl chloride	
#21	40	2.36	1	0.42	irregular fragment	~5 µm	blue	acrylonitrile butadiene styrene	
#22	41	5.47	0	0					
#23	35	3.82	0	0					
#24	48	2.53	5	1.98	irregular fragment	~2 µm	orange	polystyrene	
					irregular fragment	~10 µm	yellow	polyethylene	
					irregular fragment	~12 µm	transparent	polyethylene	
					irregular fragment	~4 µm	light blue	polypropylene	
					irregular fragment	~5 µm	brown	polyester	
#25	39	7.51	1	0.13	irregular fragment	~10 µm	blue	polyamide	
#26	37	5.65	2	0.35	irregular fragment	~6 µm	white/transparent	polyethylene	
					irregular fragment	~4 µm	light blue	polypropylene	
#27	31	3.06	1	0.33	irregular fragment	~5 µm	brown	polycarbonate	
#28	35	3.54	0	0					
#29	35	2.68	1	0.37	irregular fragment	~2 µm	light blue	polyethylene	
#30	47	1.84	5	2.72	irregular fragment	~7 µm	white/transparent	polyethylene	
					irregular fragment	~8 µm	yellow/brown	polyethylene	
					irregular fragment	~2 µm	white	polyethylene	
					irregular fragment	~4 µm	orange	high-density polyethylene	
					irregular fragment	~4 µm	blue	polyvinyl chloride	
#31	49	3.85	0	0					
#32	42	4.21	0	0					
#33	45	5.10	0	0					
#34	42	1.54	0	0					

## Data Availability

Data are contained within the article.
